# Pulmonary function and chest computed tomography abnormalities 6–12 months after recovery from COVID-19: a systematic review and meta-analysis

**DOI:** 10.1186/s12931-022-02163-x

**Published:** 2022-09-06

**Authors:** Jong Hyuk Lee, Jae-Joon Yim, Jimyung Park

**Affiliations:** 1 Department of Radiology, Seoul National University Hospital, Seoul, South Korea; 2 Division of Pulmonary and Critical Care Medicine, Department of Internal Medicine, Seoul National University College of Medicine, Seoul, South Korea; 3 Division of Pulmonary and Critical Care Medicine, Department of Internal Medicine, Seoul National University Hospital, 101, Daehak-ro, Jongno-gu, Seoul, 03080 South Korea

**Keywords:** COVID-19, Complications, Respiratory Function Tests, Computed tomography, Meta-analysis

## Abstract

**Background:**

Some coronavirus disease 2019 (COVID-19) survivors experience prolonged and varying symptoms, a condition termed post-acute COVID-19 syndrome (PACS). However, the prevalence of chronic pulmonary sequelae of PACS during long-term follow-up remains unclear. Several studies have examined this issue and reported heterogeneous results.

**Methods:**

We conducted a systematic review and meta-analysis using a random-effects model to estimate the pooled prevalence of the pulmonary sequelae of COVID-19, as demonstrated by pulmonary function testing (PFT) and chest computed tomography (CT) performed at least 6 months after initial infection. PubMed, Embase, and Cochrane Library databases were searched from January 1, 2020 to December 31, 2021 to identify related studies. We investigated whether the prevalence of pulmonary sequelae decreased over time and attempted to identify the factors associated with their development by performing multiple subgroup and meta-regression analyses.

**Results:**

Of the 18,062 studies identified, 30 met our eligibility criteria. Among these studies, 25 and 22 had follow-up PFT and chest CT data, respectively. The follow-up durations were approximately 6 and 12 months in 18 and 12 studies, respectively. Impaired diffusion capacity was the most common abnormality on PFT (pooled prevalence 35%, 95% confidence interval [CI] 30–41%) with a prevalence of 39% (95% CI 34–45%) and 31% (95% CI 21–40%) in the 6-month and 12-month follow-up studies, respectively (*P* = 0.115). Restrictive pulmonary dysfunction evident as reduced forced vital capacity was less frequent (pooled prevalence 8%, 95% CI 6–11%); however, its prevalence was lower in the 12-month follow-up studies than in the 6-month follow-up studies (5% [95% CI 3–7%] vs. 13% [95% CI 8–19%], *P* = 0.006). On follow-up chest CT, the pooled prevalence of persistent ground-glass opacities and pulmonary fibrosis was 34% (95% CI 24–44%) and 32% (95% CI 23–40%), respectively, and the prevalence did not decrease over time. As every meta-analysis showed significant between-study heterogeneity, subgroup and meta-regression analyses were performed to identify potential effect modifiers; the severity of index infection was associated with the prevalence of impaired diffusion capacity and pulmonary fibrosis.

**Conclusions:**

A substantial number of COVID-19 survivors displayed pulmonary sequelae as part of PACS. Except for restrictive pulmonary dysfunction, the prevalence of these sequelae did not decrease until 1 year after initial infection. Considering the association between the severity of acute COVID-19 and risk of pulmonary sequelae, patients who recover from severe COVID-19 require close respiratory follow-up.

*Systematic review registration number* PROSPERO CRD42021234357

**Supplementary Information:**

The online version contains supplementary material available at 10.1186/s12931-022-02163-x.

## Background

Since its first detection in Wuhan, China, in December 2019, coronavirus disease 2019 (COVID-19), which is caused by severe acute respiratory syndrome coronavirus 2 (SARS-CoV-2), has become a worldwide threat [1]. Over recent years, more than 500 million people have been diagnosed with COVID-19 worldwide, resulting in more than 6 million deaths [2].

The successful development of COVID-19 vaccines and the advent and rapid spread of the omicron variant of SARS-CoV-2 have provided immunity to a large portion of the population either by vaccination or natural infection [3]. Hence, the fear of pandemic has been fading recently; however, COVID-19 may not end and eventually become an endemic. Currently, the disease severity and mortality rate of COVID-19 are decreasing [4], but an extremely large number of COVID-19 survivors could be a problem in the future. Some COVID-19 survivors experience prolonged and varying symptoms, a condition termed “post-acute COVID-19 syndrome (PACS)” or “long COVID” [5, 6].

While the definition of PACS is changing and evolving, it is usually divided into two categories: subacute (symptoms and signs persisting 4–12 weeks after acute COVID-19) and chronic (symptoms and signs persisting beyond 12 weeks after acute COVID-19) [5, 6]. The most common symptom of PACS is fatigue; however, respiratory symptoms such as dyspnea and cough also have debilitating effects on survivors [7]. Furthermore, there is a concern that pulmonary fibrosis can develop as a sequela [8]. Long-term sequelae after viral pneumonia have been documented in patients infected by other strains of the coronavirus family, including SARS-CoV-1 (SARS) and Middle East respiratory syndrome coronavirus (MERS) [9–11].

Some experts recommend serial follow-up with pulmonary function testing (PFT) and chest computed tomography (CT) for the investigation of pulmonary sequelae, especially until 12 months after acute COVID-19 [12]. Several studies have addressed the COVID-19 related pulmonary sequelae, with results being reported from approximately 1 year after the beginning of the COVID-19 outbreak; however, most of these studies had follow-up durations of only 3–6 months [13–17]. These studies demonstrated impaired diffusion capacity as the most common pulmonary sequelae.

Given the potential for the lungs to recover over time [18], the pulmonary sequelae of PACS may also improve over time. The results from studies with longer follow-up durations of up to 1 year have only recently become available [19, 20]. However, the heterogeneity in study designs and settings between different studies makes it difficult to draw conclusions. Therefore, we conducted a systematic review and meta-analysis to pool the available data from studies and estimate the prevalence of the chronic pulmonary sequelae of PACS persisting 6–12 months after acute COVID-19. Particularly, we focused on the prevalence of abnormalities demonstrated by objective tools of pulmonary assessment, including PFT and chest CT.

## Methods

### Study protocol and outcomes

This study was conducted in accordance with the Meta-analysis Of Observational Studies in Epidemiology (MOOSE) guidelines [21]. The primary outcome of this study was the prevalence of chronic pulmonary sequelae of PACS evident on PFT and chest CT 6–12 months after acute COVID-19. First, we estimated the prevalence of pulmonary abnormalities on PFT and chest CT. Second, we examined whether the prevalence of these findings had decreased over time. Third, we evaluated potential factors that could be associated with the development of such chronic sequelae. The study protocol was registered in PROSPERO (CRD42021234357).

### Eligibility criteria

The inclusion criteria were as follows: (1) studies that included patients who had recovered from acute COVID-19 and had undergone follow-up for at least 6 months after index infection; (2) prospective cohort studies; and (3) studies that included objective pulmonary assessment results (PFT and/or chest CT). “Index infection” was defined as acute infection by SARS-CoV-2, as confirmed using real-time RT-PCR. Case reports, small case series, and meeting abstracts lacking detailed data were excluded. Retrospective studies and studies that included only patients with critical COVID-19 were also excluded to minimize selection bias.

### Information source and search strategy

Two experienced investigators (JHL and JP) performed the systematic literature search using the PubMed, EMBASE, and Cochrane Library databases. Studies published between January 1, 2020 and December 31, 2021 were identified. We also manually checked the reference lists of relevant articles. Details of the search strategy are presented in Additional file 1: Appendix S1.

### Study selection

Study selection was performed according to the Preferred Reporting Items of Systematic reviews and Meta-Analyses (PRISMA) flowchart [22]. After removing duplicates, two investigators (JHL and JP) independently reviewed the titles and abstracts of the identified studies to determine whether they should be included for further full-text reviews. After a full-text review, studies that met our full eligibility criteria were finally selected. Any disagreement regarding study selection was resolved by discussion with a third author (JJY).

### Data extraction and data items

Data on the prespecified variables were extracted from the selected studies. The extracted data included the study design, study region, study period, follow-up interval after acute COVID-19, inclusion criteria of the study, demographics of the included patients, sample size, data on the severity of acute COVID-19, details of the patient assessment tools used (PFT and/or chest CT) during follow-up, and results of those assessments. The study authors were contacted to obtain additional information if needed.

Disease severity was determined according to the current global guideline with slight modifications for the purpose of our study [23]. Because the included studies did not employ the universal disease severity criteria and as some did not clearly present data on disease severity using the predefined criteria, patients who required supplemental oxygen therapy were considered as having severe COVID-19. Patients who required intensive care unit admission and/or mechanical ventilation were considered as having critical COVID-19.

Regarding PFT data, we extracted the percent predicted values for the diffusion capacity for carbon monoxide (DLCO), forced vital capacity (FVC), and total lung capacity (TLC). Subsequently, we obtained data on the prevalence of impairment of these lung function parameters using a cutoff of 80% of the predicted values or lower limit of normal (LLN), as presented in the searched studies.

For the searched studies with follow-up chest CT data, the prevalence of pulmonary fibrosis as a sequela of COVID-19 was estimated. Although most studies clearly presented the number of patients who developed pulmonary fibrosis, some studies only reported the prevalence of various individual CT findings, including ground-glass opacity (GGO), reticulation, traction bronchiectasis, interstitial thickening, parenchymal band, and consolidation. For studies in which pulmonary fibrosis was not clearly defined, one author (JHL, 10 years of experience in thoracic radiology) adjudicated the presence of pulmonary fibrosis based on the Fleischner Society’s nomenclature and definition [24]. CT findings such as reticular opacity, architectural distortion, traction bronchiectasis, and honeycombing were considered indicative of pulmonary fibrosis. Conversely, GGO and consolidation were considered unrelated to pulmonary fibrosis. In addition to pulmonary fibrosis, we estimated the prevalence of persistent GGO as it is the most common finding in the acute phase [25]. The proportion of patients without any residual abnormal findings (complete radiologic resolution) on follow-up CT was also reviewed.

### Risk of bias assessment

We assessed the risk of bias of included studies using a tool developed and validated for prevalence studies [26]. The detailed items of questions included in this tool are described in Additional file 1: Appendix S2. Briefly, four and six items were used to evaluate the external and internal validity, respectively. Because the studies of interest were those reporting the results of objective and direct assessments of patients (PFT and/or chest CT), the overall risk of bias was mostly determined according to the external validity, rather than the internal validity, of individual studies. Particularly, we assessed whether the included patients could represent the entire target population of patients with COVID-19 in the real world.

### Statistical analysis

Considering the heterogeneity among the included studies, the DerSimonian and Laird random-effects model was used for our meta-analysis [27]. First, the results of PFT were pooled using the “metan” command [28]. For studies providing summary statistics as the median and interquartile range (IQR), rather than the mean and standard deviation (SD), the mean and SD values were estimated [29]. Subsequently, our primary study outcome, namely the pooled prevalence and 95% confidence interval (CI) of impaired lung function (less than 80% of predicted values or LLN), was calculated for each parameter (DLCO, FVC, and TLC) using the “metaprop” command [30]. The pooled prevalence of chest CT abnormalities (pulmonary fibrosis and persistent GGO) was also analyzed.

To compare the prevalence of pulmonary sequelae according to the follow-up duration, we performed subgroup analyses. Additional subgroup analyses were performed according to the risk of bias of the included studies and proportion of patients with severe acute COVID-19. Given the significant between-study heterogeneity, meta-regression analyses were also performed to identify potential factors associated with the development of pulmonary sequelae, using the “metareg” command [31]. Candidate variables for the meta-regression included age, cigarette smoking history, and the severity of acute COVID-19.

To assess the association between the development of pulmonary sequelae and severity of acute COVID-19, studies that reported the prevalence of pulmonary sequelae according to severity of index infection (severe vs. non-severe or critical vs. non-critical) were identified. Using these studies, odds ratios (ORs) were calculated by estimating the risk of development of pulmonary sequelae according to initial disease severity, and these data were subsequently used to calculate the pooled OR. For all meta-analyses, the heterogeneity among the studies was statistically investigated using the Q-test and *I*^2^ index. We visually inspected funnel plots and employed Egger’s regression to assess possible publication biases [32]. All statistical analyses were performed using the STATA software version 14.0 (StataCorp LP, College Station, TX, USA).

## Results

### Search findings and study characteristics

**Fig. 1:**
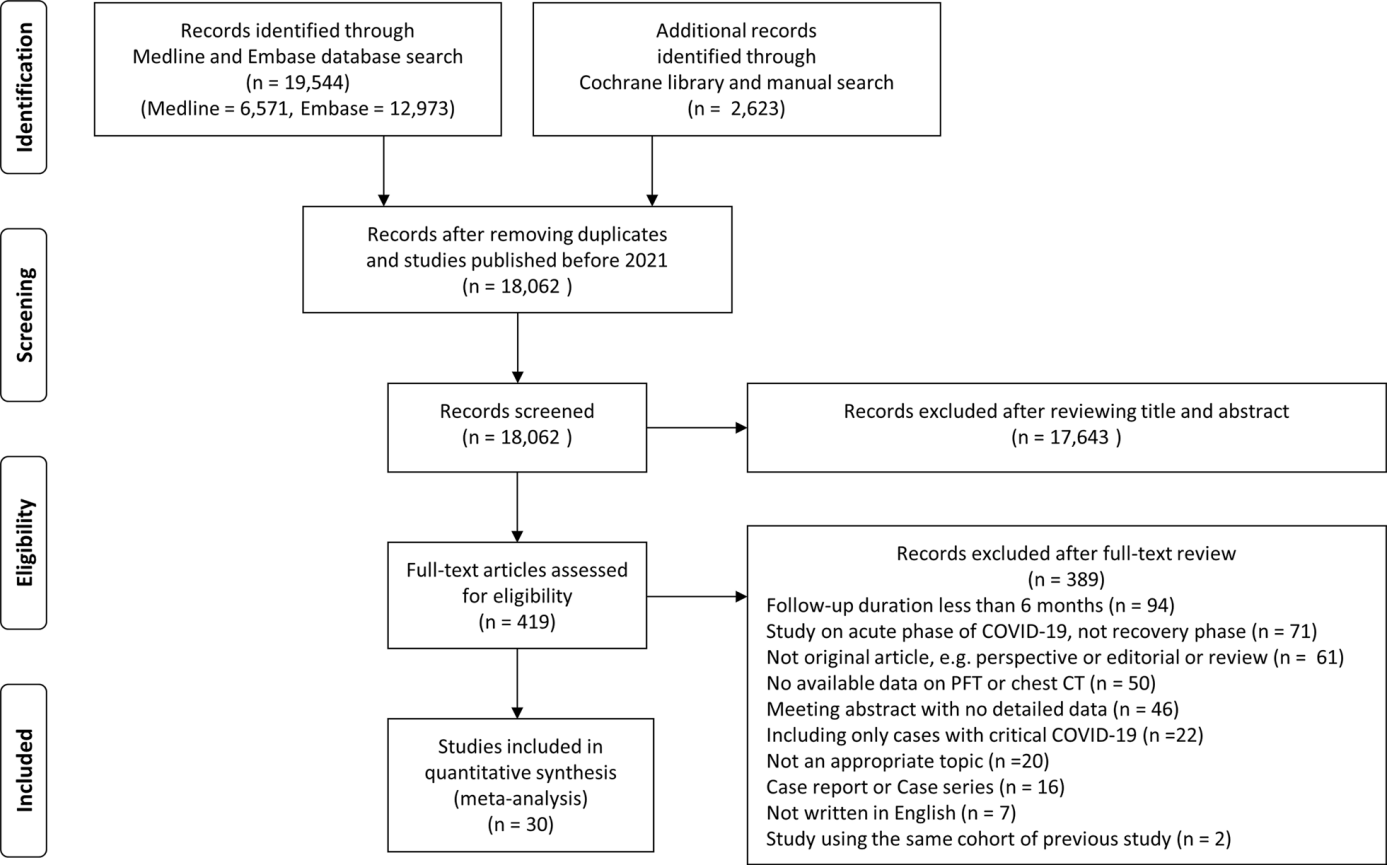
Preferred Reporting Items of Systematic reviews and Meta-Analyses (PRISMA) flowchart for this systematic review and meta-analysis

A total of 18,062 studies were identified through the literature search after removing duplicates. The full texts of 419 studies were reviewed, and 30 were finally included in our meta-analysis (Fig. 1) [19, 20, 33–60]. The details of the included studies are presented in Table 1 and Additional file 1: Table S1. The studies were mainly conducted in Asia (16, 53.3%) and Europe (13, 43.3%), and all studies were prospective cohort studies. Among the 30 studies, 25 (83.3%) had follow-up PFT data [19, 20, 33–35, 37, 38, 40–44, 46–49, 51, 53–60], and 22 (73.3%) had follow-up chest CT data [19, 20, 34–36, 38, 39, 42–48, 50–53, 56, 57, 59, 60]. The eligibility criteria varied between studies, but most studies included patients with confirmed COVID-19 who were successfully treated and discharged from the study hospitals. In most studies, PFT and chest CT were performed for every included patient as much as possible, and the proportion of patients undergoing PFT and chest CT was 88.0% and 79.7%, respectively. However, in some studies, chest CT was selectively performed (e.g., patients with abnormal results on PFT) [19, 43, 50, 51, 53].

**Table 1:** Patient characteristics of the included studies

Study	Total patients included (n)	Patients with PFT (n, %)	Patients with chest CT (n, %)	Follow-up interval ( months)	Mean age (year)	Male sex (%)	Ever smoker	Severe COVID-19	Critical COVID-19
Aparisi A	70	70 (100%)	0	6	55	36	N.A	N.A	N.A
Bardakci MI	65	59 (91%)	60 (92%)	6	N.A	75	46%	100%	12%
Bellan M	200	196 (98%)	190 (95%)	12	61	61	45%	72%	33%
Caruso D	118	0	118 (100%)	6	65	47	43%	N.A	74%
Cassar MP	46	46 (100%)	0	6	55	63	37%	59%	50%
Chen Y	41	41 (100%)	41 (100%)	12	49	59	10%	39%	N.A
Dai S	50	0	45 (90%)	6	48	50	N.A	22%	N.A
Dorelli G	28	28 (100%)	0	6	56	79	32%	57%	36%
Faverio P	312	312 (100%)	0	6	61	73	26%	100%	77%
Han X	114	104 (91%)	114 (100%)	6	54	70	14%	100%	25%
Hellemons ME	92	43 (47%)	46 (50%)	6	58	63	N.A	92%	66%
Huang C	1733	349 (20%)	353 (20%)	6	56	52	8%	75%	25%
Huang L	1276	244 (19%)	118 (9%)	12	58	53	18%	77%	29%
Li Y	141	0	141 (100%)	6	59	63	11%	N.A	7%
Liao T	303	303 (100%)	256 (84%)	12	40	19	3%	63%	N.A
Liu M	41	41 (100%)	41 (100%)	6	50	54	N.A	37%	17%
Liu T	594	486 (82%)	486 (82%)	12	61	46	13%	89%	11%
Milanese M	135	135 (100%)	0	6	59	67	21%	100%	36%
Nabahati M	173	0	62 (36%)	6	54	33	N.A	54%	N.A
Orzes N	40	40 (100%)	21 (53%)	6	58	78	N.A	67%	35%
Pan F	209	0	209 (100%)	12	49	45	2%	49%	11%
Safont B	313	313 (100%)	226 (72%)	6	61	59	44%	100%	28%
Shah AS	73	73 (100%)	0	6	63	60	32%	N.A	N.A
Staudt A	101	101 (100%)	0	12	59	58	35%	N.A	20%
Vijayakumar B	32	26 (81%)	32 (100%)	12	62	66	59%	100%	44%
Wu Q	54	53 (98%)	48 (89%)	6	48	59	N.A	43%	N.A
Wu X	83	83 (100%)	83 (100%)	12	59	57	0%	100%	55%
Yan X	119	119 (100%)	0	12	53	41	28%	24%	N.A
Zhao Y	94	70 (74%)	94 (100%)	12	48	57	7%	46%	2%
Zhou F	120	116 (97%)	97 (81%)	12	52	41	13%	13%	N.A

COVID-19, coronavirus disease 2019; CT, computed tomography; PFT, pulmonary function test

### Study population

A total of 6770 patients (mean age, 56 ± 6 years) were included, of which 56.1% were men. Smoking history data were presented in 23 studies, and approximately 24 ± 17% of the patients were ever smokers. The severity of acute COVID-19 was highly variable. The proportions of patients with severe and critical COVID-19 were 67 ± 28% and 33 ± 22%, respectively. However, some studies did not present data on the severity of index infection [33, 54]. The included studies could be approximately classified into two groups according to their follow-up durations: 18 studies with a 6-month follow-up [33, 34, 36, 37, 39–45, 47, 49–51, 53, 54, 57] and 12 studies with a 12-month follow-up [19, 20, 35, 38, 46, 48, 52, 55, 56, 58–60] (Table 1).

### Deterioration of lung function on follow-up PFT

**Fig. 2:**
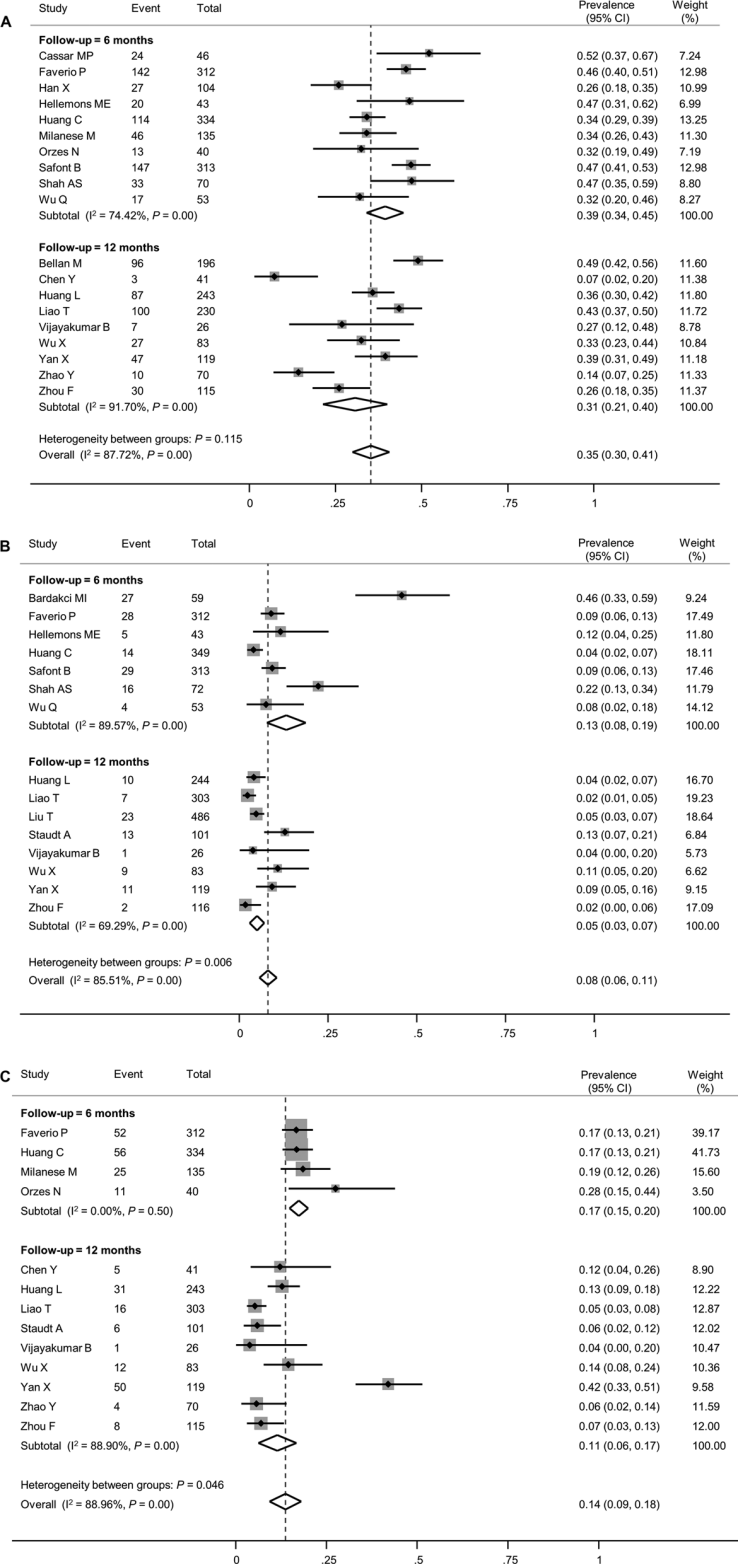
Forest plot presenting the pooled estimate of prevalence of the lung function impairment in patients who had recovered from COVID-19. **A** Diffusion capacity for carbon monoxide (DLCO), **B** Forced vital capacity (FVC), **C** Total lung capacity (TLC)

The most common abnormality on follow-up PFT was impaired diffusion capacity. The mean percent predicted DLCO was estimated to be 81.5% (95% CI 78.0–84.9%, Additional file 1: Figure S1A). The pooled estimate of the prevalence of impaired diffusion capacity was 35% (95% CI 30–41%), with considerable statistical heterogeneity (*I*^2^ = 87.7%, *P* < 0.01, Fig. 2A). Although the prevalence seemed numerically lower in the 12-month follow-up studies (31%, 95% CI 21–40%) than in the 6-month follow-up studies (39%, 95% CI 34–45%), it did not reach statistical significance (*P* = 0.115, Table 2).

**Table 2:** Subgroup analysis to explore heterogeneity in the estimate of the pooled prevalence of pulmonary function abnormalities

	Studies (k)	Patients (n)	Pooled prevalence (95% CI)	*I*^2^ (%)	*P* value
Impaired diffusion capacity	19	2573	0.35 (0.30–0.41)	87.72	
Follow-up interval after initial presentation (months)					0.115
6	10	1450	0.39 (0.34–0.45)	74.42	
12	9	1123	0.31 (0.21–0.40)	91.70	
Risk of bias of study					0.931
Low-to-moderate	12	1803	0.35 (0.28–0.43)	91.31	
Moderate-to-high	7	770	0.36 (0.29–0.42)	69.20	
Proportion of patients with severe COVID-19 (%)^a^					0.015
≥ 50	13	2105	0.39 (0.35–0.44)	73.90	
< 50	5	398	0.24 (0.12–0.35)	88.62	
Reduced forced vital capacity	15	2679	0.08 (0.06–0.11)	85.51	
Follow-up interval after initial presentation (months)					0.006
6	7	1201	0.13 (0.08–0.19)	89.57	
12	8	1478	0.05 (0.03–0.07)	69.29	
Risk of bias of study					0.033
Low-to-moderate	10	2219	0.06 (0.04–0.08)	78.97	
Moderate-to-high	5	550	0.15 (0.07–0.24)	87.16	
Proportion of patients with severe COVID-19 (%)^a^					0.544
≥ 50	10	2218	0.08 (0.05–0.10)	86.82	
< 50	3	288	0.06 (0.00–0.11)	75.23	

COVID-19, coronavirus disease 2019

^a^Some of the studies included in the overall meta-analysis did not report data on the disease severity of patients and were therefore not included in the subgroup analysis

Restrictive pulmonary dysfunction, as demonstrated by reduced FVC and/or TLC, was less frequent than impaired diffusion capacity. The mean percent predicted values of FVC and TLC were estimated to be 95.2% (95% CI 91.8–98.7%, Additional file 1: Figure S1B) and 96.3% (95% CI 90.5–102.2%, Additional file 1: Figure S1C), respectively. The pooled estimates of the prevalence of reduced FVC and TLC were 8% (95% CI 6–11%) and 14% (95% CI 9–18%), respectively (Fig. 2B and C), although the between-study heterogeneity was significant. For both FVC and TLC, the impairment was less prevalent at the 12-month follow-up than at the 6-month follow-up (5% vs. 13% for FVC [*P* = 0.006] and 11% vs. 17% for TLC [*P* = 0.046]). The funnel plot of FVC exhibited significant asymmetry, suggesting a possible publication bias (Additional file 1: Figure S2B), while those of DLCO and TLC did not (Additional file 1: Figure S2A and C).

**Fig. 3:**
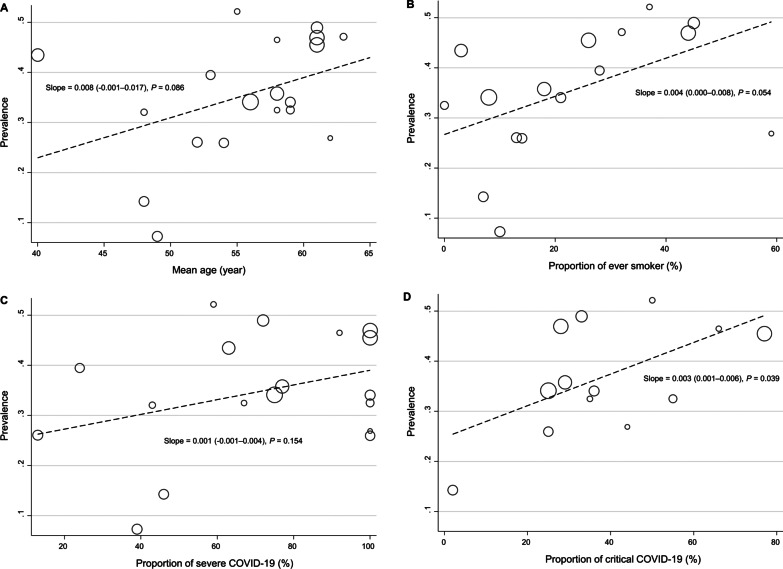
Meta-regression plots presenting the effect of patient characteristics on the prevalence of impaired diffusion capacity. **A** Age, **B** Smoking history, **C** Proportion of patients with severe COVID-19, **D** Proportion of patients with critical COVID-19

Several meta-regression analyses were performed to evaluate the factors contributing to between-study heterogeneity in the prevalence of impaired diffusion capacity and reduced FVC and TLC. In the meta-regression, only the proportion of patients with critical acute COVID-19 was associated with a higher prevalence of impaired diffusion capacity (Fig. 3D). Among other variables, age and smoking history showed only marginal associations (Fig. 3A and B). Likewise, when subgroup analyses were performed by dividing the included studies into two groups according to the proportion of patients with severe acute COVID-19 using a 50% cutoff value, the prevalence of impaired diffusion capacity was higher in studies that included 50% or more patients with severe acute COVID-19 (Table 2). For FVC and TLC, no definite effect modifier was identified in the meta-regression analysis (Additional file 1: Figures S3 and S4).

**Fig. 4:**
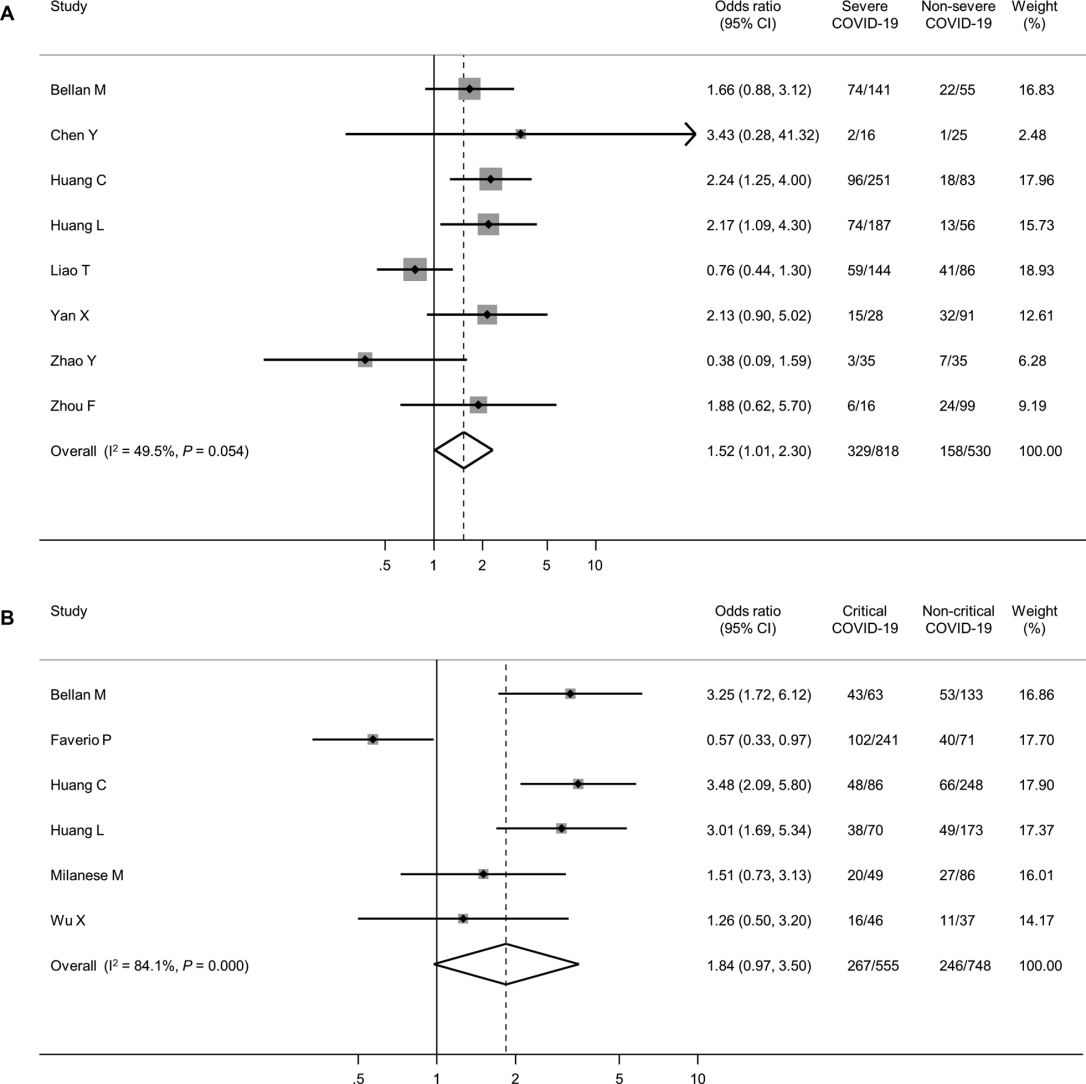
Forest plot presenting the association between the severity of index COVID-19 and the impairment of diffusion capacity. **A** Severe vs. Non-severe, **B** Critical vs. Non-critical

Eleven studies reported the prevalence of impaired diffusion capacity according to the severity of acute COVID-19 [19, 20, 35, 38, 41, 44, 46, 49, 58–60]. Using these studies, the pooled OR for impaired diffusion capacity according to disease severity (severe vs. non-severe or critical vs. non-critical) was estimated. Patients with severe COVID-19 were more likely to exhibit impaired diffusion capacity (pooled OR 1.52, 95% CI 1.01–2.30, Fig. 4A). Similarly, patients with critical COVID-19 were more likely to exhibit impaired diffusion capacity (pooled OR 1.84, 95% CI 0.97–3.50, Fig. 4B). For FVC and TLC, few studies described the prevalence of impairment based on the severity of acute COVID-19; thus, a meta-analysis could not be performed.

### Radiologic sequelae on follow-up CT

Of the 22 studies reporting the results of follow-up chest CT, we were able to pool the prevalence of pulmonary fibrosis from 18 studies [19, 20, 34, 36, 39, 42–48, 50, 52, 53, 57, 59, 60]. Of these studies, the prevalence of pulmonary fibrosis was clearly described in 10 studies according to CT interpretations by the study authors [36, 42, 43, 46–48, 50, 53, 59, 60], whereas we estimated the prevalence based on individual CT findings in the remaining 8 studies, as described above [19, 20, 34, 39, 44, 45, 52, 57].

**Fig. 5:**
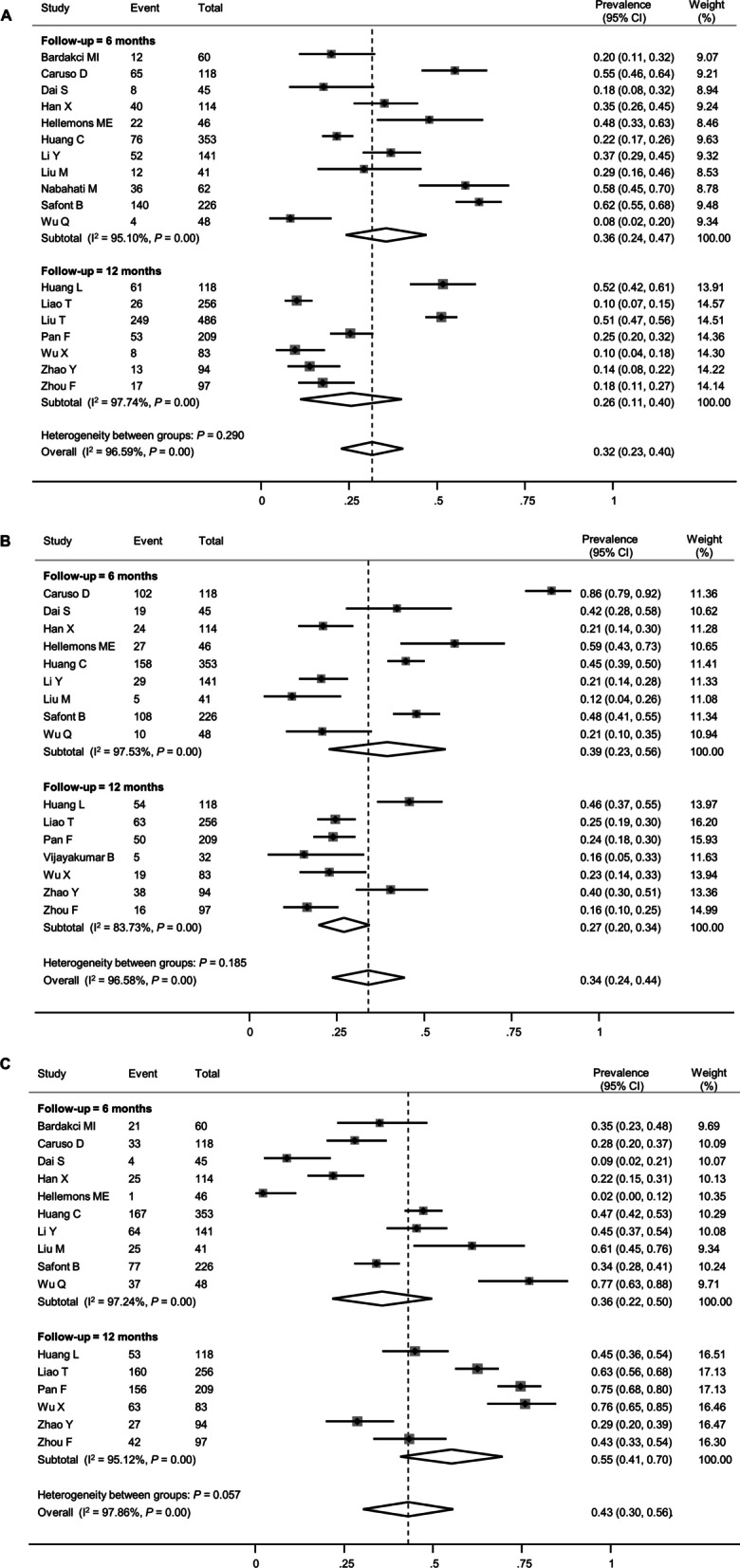
Forest plot presenting the pooled estimate of the prevalence of chest CT findings in patients who had recovered from COVID-19. **A** Pulmonary fibrosis, **B** Ground-glass opacity, **C** Normal (complete resolution)

**Fig. 6:**
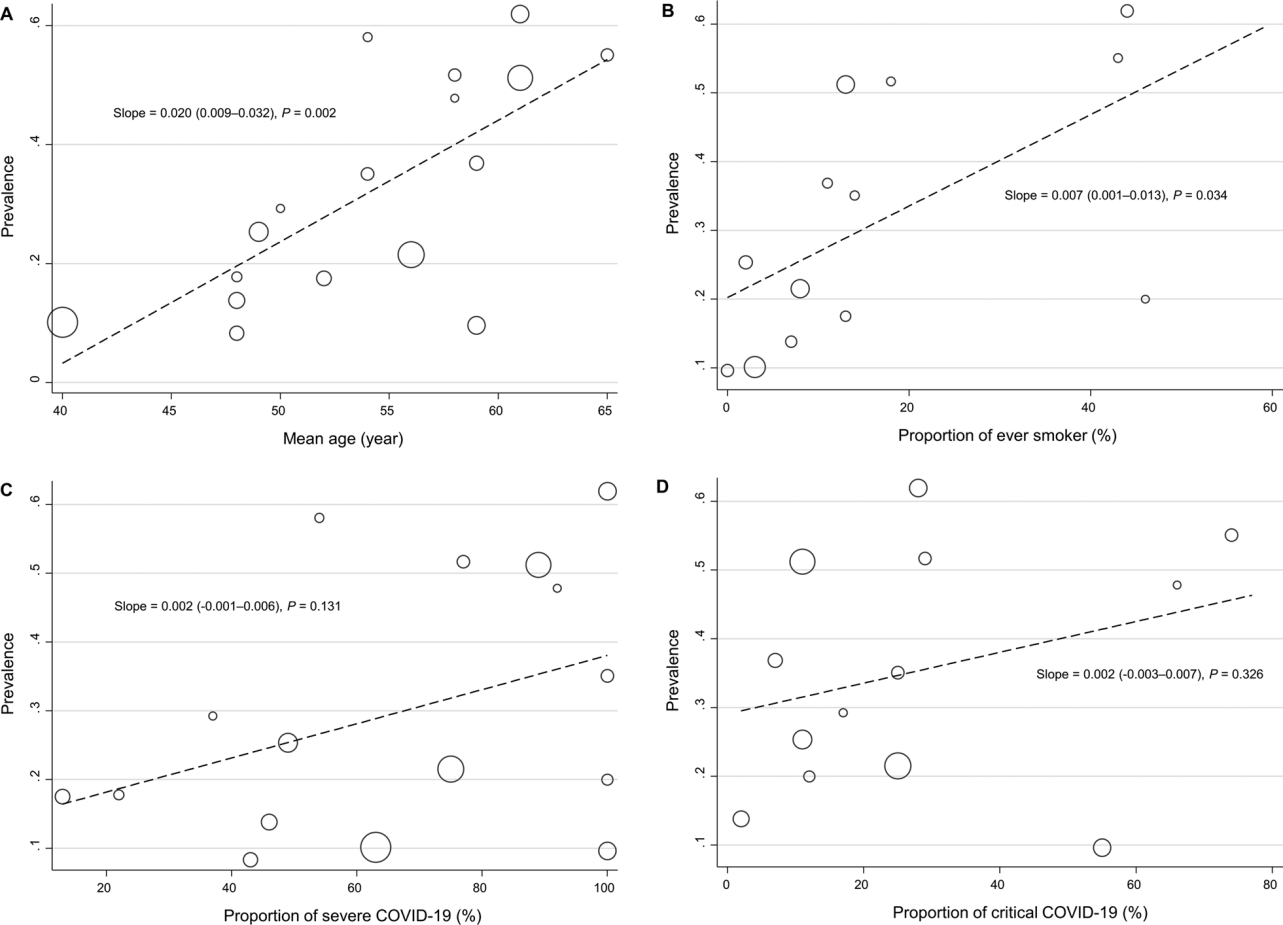
Meta-regression plots presenting the effects of patient characteristics on the prevalence of pulmonary fibrosis. **A** Age, **B** Smoking history, **C** Proportion of patients with severe COVID-19, **D** Proportion of patients with critical COVID-19

The pooled prevalence of pulmonary fibrosis was 32% (95% CI 23–40%, Fig. 5A). However, considerable statistical heterogeneity was observed (*I*^2^ = 96.6%, *P* < 0.01). The follow-up duration did not have an impact on the prevalence of pulmonary fibrosis (36% at the 6-month follow-up vs. 26% at the 12-month follow-up, *P* = 0.290). In another subgroup analysis, studies that included 50% or more patients with severe acute COVID-19 showed a higher pooled prevalence of pulmonary fibrosis (36% vs. 18%, *P* = 0.014, Table 3). In addition, meta-regression analyses showed that old age and a history of cigarette smoking were associated with a higher prevalence of pulmonary fibrosis (Fig. 6A and B).

**Table 3:** Subgroup analysis to explore heterogeneity in the estimate of the pooled prevalence of pulmonary fibrosis

	Studies (k)	Patients (n)	Pooled prevalence (95% CI)	*I*^2^ (%)	*P* value
Overall	18	2597	0.32 (0.23–0.40)	96.59	
Follow-up interval after initial presentation (months)					0.290
6	11	1254	0.36 (0.24–0.47)	95.10	
12	7	1343	0.26 (0.11–0.40)	97.74	
Risk of bias of study					0.966
Low-to-intermediate	9	1880	0.31 (0.18–0.44)	97.80	
Intermediate-to-high	9	717	0.32 (0.19–0.44)	94.05	
Proportion of patients with severe COVID-19 (%)^a^					0.014
≥ 50	10	1804	0.36 (0.23–0.50)	73.90	
< 50	6	534	0.18 (0.12–0.24)	88.62	
Pulmonary fibrosis definition					0.102
Defined clearly in the original article	10	1540	0.38 (0.23–0.52)	97.63	
Estimated by the authors	8	1057	0.24 (0.15–0.33)	91.59	

COVID-19, coronavirus disease 2019

^a^Some studies included in the overall meta-analysis did not report data on the disease severity of patients and were therefore not included in the subgroup analysis

Persistent GGO was also a common abnormality on follow-up CT, with a pooled prevalence of 34% (95% CI 24–44%, Fig. 5B). The prevalence of persistent GGO was not lower at the 12-month follow-up than at the 6-month follow-up (27% vs. 39%, *P* = 0.185). The proportion of patients with no residual abnormalities on follow-up CT, which indicates complete radiologic resolution, was also estimated. Overall, 43% of the patients (95% CI 30–56%) displayed no residual chest CT abnormalities (Fig. 5C). This proportion seemed to be higher at the 12-month follow-up than at the 6-month follow-up (55% vs. 36%, *P* = 0.057), although the difference was statistically marginal. Funnel plots did not display asymmetry in the meta-analysis of CT findings (Additional file 1: Figure S5).

### Risk of bias assessment

The assessment of the risk of bias of the included studies is summarized in Additional file 1: Table S2. Among the 30 included studies, 17 studies were regarded as having a low-to-moderate risk of bias and 13 studies were regarded as having a moderate-to-high risk of bias. However, the risk of bias of individual studies was not associated with the estimated prevalence of impaired diffusion capacity and pulmonary fibrosis on chest CT (Tables 2 and 3). However, for FVC, studies with a moderate-to-high risk of bias demonstrated a higher prevalence of reduced FVC than those with a low-to-moderate risks of bias (15% vs. 6%, *P* = 0.033).

## Discussion

Given the large number of patients affected by COVID-19 during the pandemic, the long-term consequences of COVID-19 are receiving increasing attention. The persistence of symptoms and signs that continue or develop 12 weeks after acute COVID-19 is described as PACS if they cannot be explained by an alternative diagnosis [5, 61]. PACS can manifest with a broad spectrum of symptoms and signs and involve almost every system, including the pulmonary, hematologic, cardiovascular, and neuropsychiatric systems. The most common symptoms are fatigue, dyspnea, and sleep disturbance; however, the prevalence of these symptoms is highly variable among studies [7, 62].

Although it can be assumed that the sequelae of acute infection may improve over time, many patients are reported to experience subjective symptoms, including fatigue and dyspnea, even 1 year after index infection. In particular, a recent meta-analysis showed that the prevalence of persistent dyspnea did not significantly decrease until 1 year after acute COVID-19 [62]. However, dyspnea is a complex subjective symptom that can result from various pathological mechanisms [63]. Thus, it cannot always be explained by the extent of pulmonary parenchymal abnormalities. For example, some studies using cardiopulmonary exercise tests have demonstrated that persistent dyspnea after COVID-19 was mostly due to deconditioning or dysfunctional breathing such as hyperventilation [64, 65]. Because all COVID-19 survivors with persistent dyspnea cannot undergo resource-consuming examinations such as PFT and chest imaging, it is imperative to assess the real prevalence of abnormities demonstrated by such objective tools to comprehend the clinical impact of PACS more accurately and tailor the patients’ follow-up plans more appropriately.

Several attempts have been made to evaluate COVID-19 survivors via follow-up PFT and/or chest CT. The results of these studies have been published since the end of 2020, revealing impaired diffusion capacity as the most common abnormality [13, 15, 66, 67]. Two meta-analyses were performed to pool the data regarding follow-up PFT and chest CT of COVID-19 survivors [68, 69]. However, the included studies mostly had limited follow-up durations of 6 months or less. To clarify the longer-term sequelae of COVID-19, relevant studies with longer follow-up durations of 6–12 months have been increasingly conducted. In this study, we pooled only such longer follow-up studies. Thus, we were able to address the chronic pulmonary sequelae of PACS more clearly and investigate whether their prevalence decreased over time by comparing the 6-month and 12-month follow-up studies.

Overall, abnormal diffusion, as indicated by impaired diffusion capacity, was the most common abnormality with a pooled prevalence of 35%. Although the 12-month follow-up studies seemed to show a lower prevalence of impaired diffusion capacity than the 6-month follow-up studies, the difference was not statistically significant. Similarly, previous studies on SARS and MERS demonstrated that a considerable proportion of survivors had persistent impairment in diffusion capacity 1 year after infection [70, 71].

In our study, several subgroup and meta-regression analyses suggested an association between the initial severity of acute COVID-19 and prevalence of impaired diffusion capacity at long-term follow-up. This association has also been reported in previous studies with shorter follow-up durations of less than 6 months [13, 14]. Therefore, although the persistent presence of subjective dyspnea or cough does not always correlate with the severity of index infection [19, 72], patients who recovered from severe COVID-19 should be more closely followed-up with PFT.

The prevalence of restrictive pulmonary dysfunction was lower than that of impaired diffusion capacity. Reduced FVC and TLC were noted in 8% and 14% of the COVID-19 survivors, respectively. Interestingly, unlike DLCO, the prevalence of reduced FVC and TLC was lower at the 12-month follow-up than at the 6-month follow-up. FVC and DLCO are widely used parameters in the assessment of the severity of interstitial lung disease, and discrepancies between these two parameters have already been reported [73]. Theoretically, it can be assumed that whereas restrictive dysfunction due to lung parenchymal abnormalities may improve over time, sequelae related to vasculopathy may not, thereby resulting in impaired DLCO with relatively preserved FVC. The effects of COVID-19 associated vasculopathy may be important not only in the acute phase of infection but also long after recovery [74].

The most common radiographic sequela on follow-up chest CT was persistent GGO (34%) followed by fibrotic changes (32%). The prevalence of these radiographic sequelae did not differ between the 6-month and 12-month follow-up studies. However, our study was only able to assess the presence of these radiographic sequelae. The extent and severity of the radiographic changes could not be evaluated in detail. A recent study reported that although long-term CT abnormalities were common 1 year after acute COVID-19, the extent of CT lesions slowly improved on serial follow-up chest CT scans [75]. Future studies should address whether subtle residual fibrotic sequelae have long-term clinical and functional significance.

Given its expense and radiation risk, chest CT cannot be performed for every symptomatic COVID-19 survivor. In our study, in addition to the severity of acute COVID-19, patients’ demographic factors, including age and smoking status, were associated with the prevalence of pulmonary fibrosis, thus suggesting that these demographic factors should be considered when deciding whether chest CT should be performed in certain patients with PACS. A recent study also revealed that ages greater than 60 years were associated with persistent CT abnormalities at 1-year follow up [75]. However, this finding might have been confounded by the fact that older age is usually associated with more severe COVID-19 manifestations [76]. It is also difficult to interpret the effect of smoking, as it is possible for fibrotic interstitial lung abnormalities in smokers to be detected incidentally after COVID-19 [77]. Moreover, the effect of smoking on pulmonary fibrosis might have been mediated by its effect on the risk of severe COVID-19 [78].

Although we could not perform a meta-analysis due to insufficient data, a few studies have reported the association between impaired diffusion capacity and the presence of fibrotic sequelae on chest CT [79, 80]. Taking these findings together, when planning follow-ups for COVID-19 survivors, patients with persistent symptoms after recovering from more severe forms of COVID-19 may be recommended for more careful follow-ups with regular PFT, particularly with DLCO measurements. Chest CT should be considered for more selective patients, especially the elderly or those who show abnormalities on PFT.

This study has limitations. First, most meta-analyses have shown considerable statistical heterogeneity. This problem is believed to arise from the heterogeneity of the study design and patient characteristics among the included studies. Furthermore, most included studies were single-center analyses with small sample sizes. We attempted to assess the source of this heterogeneity via meta-regression and subgroup analyses. Second, although spirometry can be more easily standardized in most laboratory units, DLCO measurements are more prone to variability across different laboratory units [81]. Similarly, the presence of pulmonary fibrosis was determined by the investigators of each study; however, this could not be adjudicated in this study, which might have also contributed to the heterogeneity between the studies. Although most studies followed the Fleischner Society’s nomenclature and definition [24], between-study heterogeneity could have occurred in defining the pulmonary fibrosis. For example, some studies simply presented the proportion of patients with pulmonary fibrosis, but specific CT findings such as traction bronchiectasis or reticular opacity were not mentioned. Third, most studies did not report data on the baseline lung function of the study patients before the index infection. Therefore, some patients with underlying lung disease and impaired lung function might have been counted as exhibiting pulmonary sequelae. Additionally, most studies included hospitalized patients, and a substantial proportion of patients had severe COVID-19. Therefore, the pooled prevalence of pulmonary sequelae in our meta-analysis might have been overestimated. Fourth, there were no available studies with a follow-up duration of more than 1 year. Although a considerable number of patients demonstrated abnormalities on PFT and chest CT at their 1-year follow-up, we cannot conclude whether these pulmonary sequelae would persist or improve over longer periods. Therefore, further follow-up studies are required. Finally, most of the included studies enrolled patients in 2020 or early 2021 before the advent of the omicron variant. The number of enrolled patients with the delta variant was also likely very small. As the SARS-CoV-2 variant types can affect the risk of PACS, more studies should be conducted to compare the severity and risk of PACS among different variants.

## Conclusions

In conclusion, a substantial number of COVID-19 survivors exhibited chronic pulmonary sequelae as demonstrated by PFT and chest CT. Impaired diffusion capacity was the most common abnormality on PFT, and its prevalence was not negligible even 12 months after index infection; however, restrictive pulmonary dysfunction was less frequent, and its prevalence was lower at the 12-month follow-up than at the 6-month follow-up. Persistent GGO and pulmonary fibrosis were also common on follow-up chest CT 12 months after index infection. The severity of acute COVID-19 was associated with the risk of impaired diffusion capacity and pulmonary fibrosis. Given this association, careful respiratory follow-up is warranted, especially in patients who recover from severe COVID-19.

## Supplementary Information

**Additional file 1. Appendix 1** Literature search strategy. **Appendix 2** Tools for assessing risk of bias in this study. **Figure S1**. Forest plot presenting the pooled estimate of percent predicted values for lung function. parameters. **Figure S2**. Funnel plots presenting publication biases of studies on PFT abnormalities. **Figure S3**. Meta-regression plots presenting the effects of patient characteristics on the prevalence of reduced FVC. **Figure S5**. Meta-regression plots presenting the effects of patient characteristics on the prevalence of reduced TLC. **Figure S5**. Funnel plots presenting publication biases of studies on chest CT findings. **Table S1**. Summary of included studies. **Table S2**. Summary of risk of bias evaluation.

## Data Availability

The datasets used and/or analyzed during the current study are available from the corresponding author on reasonable request.

## References

[1] Guan WJ, Ni ZY, Hu Y, Liang WH, Ou CQ, He JX (2020). Clinical characteristics of coronavirus disease 2019 in China. N Engl J Med.

[2] World Health Organization. WHO coronavirus (COVID-19) dashboard. https://covid19.who.int/.

[3] Araf Y, Akter F, Tang YD, Fatemi R, Parvez MSA, Zheng C (2022). Omicron variant of SARS-CoV-2: genomics, transmissibility, and responses to current COVID-19 vaccines. J Med Virol.

[4] World Health Organization (2022). Severity of disease associated with Omicron variant as compared with Delta variant in hospitalized patients with suspected or confirmed SARS-CoV-2 infection.

[5] Nalbandian A, Sehgal K, Gupta A, Madhavan MV, McGroder C, Stevens JS (2021). Post-acute COVID-19 syndrome. Nat Med.

[6] Montani D, Savale L, Noel N, Meyrignac O, Colle R, Gasnier M (2022). Post-acute COVID-19 syndrome. Eur Respir Rev.

[7] Han Q, Zheng B, Daines L, Sheikh A (2022). Long-term sequelae of COVID-19: a systematic review and meta-analysis of one-year follow-up studies on post-COVID symptoms. Pathogens.

[8] Spagnolo P, Balestro E, Aliberti S, Cocconcelli E, Biondini D, Casa GD (2020). Pulmonary fibrosis secondary to COVID-19: a call to arms?. Lancet Respir Med.

[9] Zhang P, Li J, Liu H, Han N, Ju J, Kou Y (2020). Long-term bone and lung consequences associated with hospital-acquired severe acute respiratory syndrome: a 15-year follow-up from a prospective cohort study. Bone Res.

[10] Das KM, Lee EY, Singh R, Enani MA, Al Dossari K, Van Gorkom K (2017). Follow-up chest radiographic findings in patients with MERS-CoV after recovery. Indian J Radiol Imaging.

[11] Ngai JC, Ko FW, Ng SS, To KW, Tong M, Hui DS (2010). The long-term impact of severe acute respiratory syndrome on pulmonary function, exercise capacity and health status. Respirology.

[12] Raghu G, Wilson KC (2020). COVID-19 interstitial pneumonia: monitoring the clinical course in survivors. Lancet Respir Med.

[13] Bellan M, Soddu D, Balbo PE, Baricich A, Zeppegno P, Avanzi GC (2021). Respiratory and psychophysical sequelae among patients with covid-19 four months after hospital discharge. JAMA Netw Open.

[14] Guler SA, Ebner L, Aubry-Beigelman C, Bridevaux PO, Brutsche M, Clarenbach C (2021). Pulmonary function and radiological features 4 months after COVID-19: first results from the national prospective observational Swiss COVID-19 lung study. Eur Respir J.

[15] Lerum TV, Aaløkken TM, Brønstad E, Aarli B, Ikdahl E, Lund KMA (2021). Dyspnoea, lung function and CT findings 3 months after hospital admission for COVID-19. Eur Respir J.

[16] van den Borst B, Peters JB, Brink M, Schoon Y, Bleeker-Rovers CP, Schers H (2021). Comprehensive health assessment 3 months after recovery from acute coronavirus disease 2019 (COVID-19). Clin Infect Dis.

[17] Zhao YM, Shang YM, Song WB, Li QQ, Xie H, Xu QF (2020). Follow-up study of the pulmonary function and related physiological characteristics of COVID-19 survivors three months after recovery. EClinicalMedicine.

[18] Herridge MS, Moss M, Hough CL, Hopkins RO, Rice TW, Bienvenu OJ (2016). Recovery and outcomes after the acute respiratory distress syndrome (ARDS) in patients and their family caregivers. Intensive Care Med.

[19] Huang L, Yao Q, Gu X, Wang Q, Ren L, Wang Y (2021). 1-year outcomes in hospital survivors with COVID-19: a longitudinal cohort study. Lancet.

[20] Wu X, Liu X, Zhou Y, Yu H, Li R, Zhan Q (2021). 3-month, 6-month, 9-month, and 12-month respiratory outcomes in patients following COVID-19-related hospitalisation: a prospective study. Lancet Respir Med.

[21] Stroup DF, Berlin JA, Morton SC, Olkin I, Williamson GD, Rennie D (2000). Meta-analysis of observational studies in epidemiology: a proposal for reporting. Meta-analysis Of Observational Studies in Epidemiology (MOOSE) group. JAMA.

[22] Liberati A, Altman DG, Tetzlaff J, Mulrow C, Gotzsche PC, Ioannidis JP (2009). The PRISMA statement for reporting systematic reviews and meta-analyses of studies that evaluate healthcare interventions: explanation and elaboration. BMJ.

[23] World Health Organization. Clinical management of COVID-19: living guidance. https://apps.who.int/iris/handle/10665/338882. 35917394

[24] Hansell DM, Bankier AA, MacMahon H, McLoud TC, Muller NL, Remy J (2008). Fleischner Society: glossary of terms for thoracic imaging. Radiology.

[25] Zhou S, Wang Y, Zhu T, Xia L (2020). CT Features of Coronavirus Disease 2019 (COVID-19) pneumonia in 62 patients in Wuhan, China. Am J Roentgenol.

[26] Hoy D, Brooks P, Woolf A, Blyth F, March L, Bain C (2012). Assessing risk of bias in prevalence studies: modification of an existing tool and evidence of interrater agreement. J Clin Epidemiol.

[27] Sharp S, Sterne J (1997). sbe16: Meta-analysis. Stata Tech Bull.

[28] Harris RJ, Deeks JJ, Altman DG, Bradburn MJ, Harbord RM, Sterne JAC (2008). Metan: fixed- and random-effects meta-analysis. Stata J.

[29] Wan X, Wang W, Liu J, Tong T (2014). Estimating the sample mean and standard deviation from the sample size, median, range and/or interquartile range. BMC Med Res Methodol.

[30] Nyaga VN, Arbyn M, Aerts M (2014). Metaprop: a Stata command to perform meta-analysis of binomial data. Arch Public Health.

[31] Harbord RM, Higgins JPT (2008). Meta-Regression in Stata. Stata J.

[32] Egger M, Davey Smith G, Schneider M, Minder C (1997). Bias in meta-analysis detected by a simple, graphical test. BMJ.

[33] Aparisi A, Ybarra-Falcon C, Garcia-Gomez M, Tobar J, Iglesias-Echeverria C, Jaurrieta-Largo S (2021). Exercise ventilatory inefficiency in post-COVID-19 syndrome: insights from a prospective evaluation. J Clin Med.

[34] Bardakci MI, Ozturk EN, Ozkarafakili MA, Ozkurt H, Yanc U, Yildiz SD (2021). Evaluation of long-term radiological findings, pulmonary functions, and health-related quality of life in survivors of severe COVID-19. J Med Virol.

[35] Bellan M, Baricich A, Patrucco F, Zeppegno P, Gramaglia C, Balbo PE (2021). Long-term sequelae are highly prevalent one year after hospitalization for severe COVID-19. Sci Rep.

[36] Caruso D, Guido G, Zerunian M, Polidori T, Lucertini E, Pucciarelli F (2021). Post-acute sequelae of COVID-19 pneumonia: six-month chest CT follow-up. Radiology.

[37] Cassar MP, Tunnicliffe EM, Petousi N, Lewandowski AJ, Xie C, Mahmod M (2021). Symptom persistence despite improvement in cardiopulmonary health—insights from longitudinal CMR, CPET and lung function testing post-COVID-19. EClinicalMedicine.

[38] Chen Y, Ding C, Yu L, Guo W, Feng X, Yu L (2021). One-year follow-up of chest CT findings in patients after SARS-CoV-2 infection. BMC Med.

[39] Dai S, Zhao B, Liu D, Zhou Y, Liu Y, Lan L (2021). Follow-up study of the cardiopulmonary and psychological outcomes of COVID-19 survivors six months after discharge in Sichuan, China. Int J Gen Med.

[40] Dorelli G, Braggio M, Gabbiani D, Busti F, Caminati M, Senna G (2021). Importance of cardiopulmonary exercise testing amongst subjects recovering from COVID-19. Diagnostics (Basel).

[41] Faverio P, Luppi F, Rebora P, Busnelli S, Stainer A, Catalano M (2021). Six-month pulmonary impairment after severe COVID-19: a prospective multicentre follow-up study. Respiration.

[42] Han X, Fan Y, Alwalid O, Li N, Jia X, Yuan M (2021). Six-month follow-up chest CT findings after severe COVID-19 pneumonia. Radiology.

[43] Hellemons ME, Huijts S, Bek LM, Berentschot JC, Nakshbandi G, Schurink CAM (2022). Persistent health problems beyond pulmonary recovery up to 6 months after hospitalization for COVID-19: a longitudinal study of respiratory, physical, and psychological outcomes. Ann Am Thorac Soc.

[44] Huang C, Huang L, Wang Y, Li X, Ren L, Gu X (2021). 6-month consequences of COVID-19 in patients discharged from hospital: a cohort study. Lancet.

[45] Li Y, Han X, Huang J, Alwalid O, Jia X, Yuan M (2021). Follow-up study of pulmonary sequelae in discharged COVID-19 patients with diabetes or secondary hyperglycemia. Eur J Radiol.

[46] Liao T, Meng D, Xiong L, Wu S, Yang L, Wang S (2022). Long-term effects of COVID-19 on health care workers 1-year post-discharge in Wuhan. Infect Dis Ther.

[47] Liu M, Lv F, Huang Y, Xiao K (2021). Follow-up study of the chest CT characteristics of COVID-19 survivors seven months after recovery. Front Med (Lausanne).

[48] Liu T, Wu D, Yan W, Wang X, Zhang X, Ma K (2022). Twelve-month systemic consequences of coronavirus disease 2019 (COVID-19) in patients discharged from hospital: a prospective cohort study in Wuhan, China. Clin Infect Dis.

[49] Milanese M, Anselmo M, Buscaglia S, Garra L, Goretti R, Parodi L (2021). COVID-19 6 months after hospital discharge: pulmonary function impairment and its heterogeneity. ERJ Open Res.

[50] Nabahati M, Ebrahimpour S, Khaleghnejad Tabari R, Mehraeen R (2021). Post-COVID-19 pulmonary fibrosis and its predictive factors: a prospective study. Egypt J Radiol Nucl Med.

[51] Orzes N, Pini L, Levi G, Uccelli S, Cettolo F, Tantucci C (2021). A prospective evaluation of lung function at three and six months in patients with previous SARS-COV-2 pneumonia. Respir Med.

[52] Pan F, Yang L, Liang B, Ye T, Li L, Li L (2022). Chest CT patterns from diagnosis to 1 year of follow-up in patients with COVID-19. Radiology.

[53] Safont B, Tarraso J, Rodriguez-Borja E, Fernandez-Fabrellas E, Sancho-Chust JN, Molina V (2022). Lung function, radiological findings and biomarkers of fibrogenesis in a cohort of COVID-19 patients six months after hospital discharge. Arch Bronconeumol.

[54] Shah AS, Ryu MH, Hague CJ, Murphy DT, Johnston JC, Ryerson CJ (2021). Changes in pulmonary function and patient-reported outcomes during COVID-19 recovery: a longitudinal, prospective cohort study. ERJ Open Res.

[55] Staudt A, Jorres RA, Hinterberger T, Lehnen N, Loew T, Budweiser S (2022). Associations of post-acute COVID syndrome with physiological and clinical measures 10 months after hospitalization in patients of the first wave. Eur J Intern Med.

[56] Vijayakumar B, Tonkin J, Devaraj A, Philip KEJ, Orton CM, Desai SR (2022). CT lung abnormalities after COVID-19 at 3 months and 1 year after hospital discharge. Radiology.

[57] Wu Q, Zhong L, Li H, Guo J, Li Y, Hou X (2021). A follow-up study of lung function and chest computed tomography at 6 months after discharge in patients with coronavirus disease 2019. Can Respir J.

[58] Yan X, Huang H, Wang C, Jin Z, Zhang Z, He J (2021). Follow-up study of pulmonary function among COVID-19 survivors 1 year after recovery. J Infect.

[59] Zhao Y, Yang C, An X, Xiong Y, Shang Y, He J (2021). Follow-up study on COVID-19 survivors one year after discharge from hospital. Int J Infect Dis.

[60] Zhou F, Tao M, Shang L, Liu Y, Pan G, Jin Y (2021). Assessment of sequelae of COVID-19 nearly 1 year after diagnosis. Front Med (Lausanne).

[61] NICE Guideline. COVID-19 rapid guideline: managing the long term effects of COVID-19. https://www.nice.org.uk/guidance/ng188. 33555768

[62] Alkodaymi MS, Omrani OA, Fawzy NA, Shaar BA, Almamlouk R, Riaz M (2022). Prevalence of post-acute COVID-19 syndrome symptoms at different follow-up periods: a systematic review and meta-analysis. Clin Microbiol Infect.

[63] Parshall MB, Schwartzstein RM, Adams L, Banzett RB, Manning HL, Bourbeau J (2012). An official American Thoracic Society statement: update on the mechanisms, assessment, and management of dyspnea. Am J Respir Crit Care Med.

[64] Fresard I, Genecand L, Altarelli M, Gex G, Vremaroiu P, Vremaroiu-Coman A (2022). Dysfunctional breathing diagnosed by cardiopulmonary exercise testing in 'long COVID' patients with persistent dyspnoea. BMJ Open Respir Res.

[65] Skjorten I, Ankerstjerne OAW, Trebinjac D, Bronstad E, Rasch-Halvorsen O, Einvik G (2021). Cardiopulmonary exercise capacity and limitations 3 months after COVID-19 hospitalisation. Eur Respir J.

[66] Anastasio F, Barbuto S, Scarnecchia E, Cosma P, Fugagnoli A, Rossi G (2021). Medium-term impact of COVID-19 on pulmonary function, functional capacity and quality of life. Eur Respir J.

[67] Qin W, Chen S, Zhang Y, Dong F, Zhang Z, Hu B (2021). Diffusion capacity abnormalities for carbon monoxide in patients with COVID-19 at 3-month follow-up. Eur Respir J.

[68] So M, Kabata H, Fukunaga K, Takagi H, Kuno T (2021). Radiological and functional lung sequelae of COVID-19: a systematic review and meta-analysis. BMC Pulm Med.

[69] Torres-Castro R, Vasconcello-Castillo L, Alsina-Restoy X, Solis-Navarro L, Burgos F, Puppo H (2021). Respiratory function in patients post-infection by COVID-19: a systematic review and meta-analysis. Pulmonology.

[70] Hui DS, Wong KT, Ko FW, Tam LS, Chan DP, Woo J (2005). The 1-year impact of severe acute respiratory syndrome on pulmonary function, exercise capacity, and quality of life in a cohort of survivors. Chest.

[71] Park WB, Jun KI, Kim G, Choi J-P, Rhee J-Y, Cheon S (2018). Correlation between pneumonia severity and pulmonary complications in middle east respiratory syndrome. J Korean Med Sci.

[72] Fernandez-de-Las-Penas C, Guijarro C, Plaza-Canteli S, Hernandez-Barrera V, Torres-Macho J (2021). Prevalence of post-COVID-19 cough one year after SARS-CoV-2 infection: a multicenter study. Lung.

[73] Le Gouellec N, Duhamel A, Perez T, Hachulla AL, Sobanski V, Faivre JB (2017). Predictors of lung function test severity and outcome in systemic sclerosis-associated interstitial lung disease. PLoS ONE.

[74] Becker RC (2020). COVID-19-associated vasculitis and vasculopathy. J Thromb Thrombolysis.

[75] Luger AK, Sonnweber T, Gruber L, Schwabl C, Cima K, Tymoszuk P (2022). Chest CT of lung injury 1 year after COVID-19 pneumonia: The CovILD Study. Radiology.

[76] Shahid Z, Kalayanamitra R, McClafferty B, Kepko D, Ramgobin D, Patel R (2020). COVID-19 and older adults: what we know. J Am Geriatr Soc.

[77] Hatabu H, Hunninghake GM, Richeldi L, Brown KK, Wells AU, Remy-Jardin M (2020). Interstitial lung abnormalities detected incidentally on CT: a Position Paper from the Fleischner Society. Lancet Respir Med.

[78] Clift AK, von Ende A, Tan PS, Sallis HM, Lindson N, Coupland CAC (2022). Smoking and COVID-19 outcomes: an observational and Mendelian randomisation study using the UK Biobank cohort. Thorax.

[79] Han X, Fan Y, Alwalid O, Zhang X, Jia X, Zheng Y (2021). Fibrotic interstitial lung abnormalities at 1-year follow-up CT after severe COVID-19. Radiology.

[80] Faverio P, Luppi F, Rebora P, D'Andrea G, Stainer A, Busnelli S (2022). One-year pulmonary impairment after severe COVID-19: a prospective, multicenter follow-up study. Respir Res.

[81] McCormack MC (2012). Facing the noise: addressing the endemic variability in D(LCO) testing. Respir Care.

