# Supplementary material for: Pulmonary function and chest computed tomography abnormalities 6–12 months after recovery from COVID-19: a systematic review and meta-analysis
Source: Respir Res. 2022 Sep 6;23:233. doi: 10.1186/s12931-022-02163-x (PMC9446643; doi:10.1186/s12931-022-02163-x)
Supplement: Supplementary file 1 — Additional file 1. Appendix 1 Literature search strategy. Appendix 2 Tools for assessing risk of bias in this study. Figure S1. Forest plot presenting the pooled estimate of percent predicted values for lung function. parameters. Figure S2. Funnel plots presenting publication biases of studies on PFT abnormalities. Figure S3. Meta-regression plots presenting the effects of patient characteristics on the prevalence of reduced FVC. Figure S5. Meta-regression plots presenting the effects of patient characteristics on the prevalence of reduced TLC. Figure S5. Funnel plots presenting publication biases of studies on chest CT findings. Table S1. Summary of included studies. Table S2. Summary of risk of bias evaluation. [file 12931_2022_2163_MOESM1_ESM.docx]

[Additional File]

**Pulmonary function and Chest CT abnormalities 6–12 months after recovery from COVID-19: a systematic review and meta-analysis**

Jong Hyuk Lee^1^, Jae-Joon Yim^2^, Jimyung Park^3^,

^1^ Department of Radiology, Seoul National University Hospital, Seoul, South Korea

^2^ Division of Pulmonary and Critical Care Medicine, Department of Internal Medicine, Seoul National University College of Medicine, Seoul, South Korea

^3^ Division of Pulmonary and Critical Care Medicine, Department of Internal Medicine, Seoul National University Hospital, Seoul, South Korea

**Appendix 1 - Literature search strategy**

**Searched Database: PubMed, Embase, Cochrane library**

**1) PubMed search strategy**

**#1. COVID-19**

“COVID-19”[Mesh] OR “SARS-CoV-2”[Mesh]

OR

“COVID*” OR “COVID-19” OR “Coronavirus*” OR “Coronavirus disease*” OR “Coronavirus disease 2019” OR “SARS-CoV-2” OR “CoV-2” OR “SARS-CoV” OR “SARS” OR “Severe acute respiratory syndrome” OR “2019-nCoV” OR “nCoV” OR "Novel coronavirus*”

**#2. Follow-up**

“Long-COVID” OR “Post-acute COVID” OR “Post-COVID” OR "Follow-up" OR "Follow*" OR “Discharge*” OR “Recover*” OR “Surviv*” OR “Long-term” OR “Chronic” OR “Persistent” OR “Residual” OR “Sequel*” OR “Complication*” OR “Consequence*”

**#3. Chest CT / Pulmonary function test**

"Tomography, X-Ray Computed"[Mesh] OR "Respiratory Function Tests"[Mesh]

OR

“Computed tomography” OR “CT” OR “Chest CT” OR “PFT” OR “Pulmonary function” OR “Respiratory function” OR “Lung function” OR “Spirometry” OR “Spirometer” OR “Diffusing capacity” OR “Diffusion capacity” OR “Diffusing” OR “Diffusion” OR “Transfer factor” OR “Transfer”

**# Final search strategy: #1 AND #2 AND #3**

**2) Embase search strategy**

**#1. COVID-19**

'coronavirus disease 2019'/exp OR 'Severe acute respiratory syndrome coronavirus 2'/exp

OR

‘COVID*’ OR ‘COVID-19’ OR ‘Coronavirus*’ OR ‘Coronavirus disease*’ OR ‘Coronavirus disease 2019’ OR ‘SARS-CoV-2’ OR ‘CoV-2’ OR ‘SARS-CoV’ OR ‘SARS’ OR ‘Severe acute respiratory syndrome’ OR ‘2019-nCoV’ OR ‘nCoV’ OR ‘Novel coronavirus*’

**#2. Follow-up**

‘Long-COVID’ OR ‘Post-acute COVID’ OR ‘Post-COVID’ OR ‘Follow-up’ OR ‘Follow*’ OR ‘Discharge*’ OR ‘Recover*’ OR ‘Surviv*’ OR ‘Long-term’ OR ‘Chronic’ OR ‘Persistent’ OR ‘Residual’ OR ‘Sequel*’ OR ‘Complication*’ OR ‘Consequence*’

**#3. Chest CT / Pulmonary function test**

'computer assisted tomography'/exp OR 'lung function test'/exp

OR

‘Computed tomography’ OR ‘CT’ OR ‘Chest CT’ OR ‘PFT’ OR ‘Pulmonary function’ OR ‘Respiratory function’ OR ‘Lung function’ OR ‘Spirometry’ OR ‘Spirometer’ OR ‘Diffusing capacity’ OR ‘Diffusion capacity’ OR ‘Diffusing’ OR ‘Diffusion’ OR ‘Transfer factor’ OR ‘Transfer’

**# Final search strategy: #1 AND #2 AND #3**

**3) Cochrane Library search strategy**

**#1. COVID-19**

MeSH descriptor: [COVID-19] explode all trees

OR

COVID* OR COVID-19 OR Coronavirus* OR Coronavirus disease* OR Coronavirus disease 2019 OR SARS-CoV-2 OR CoV-2 OR SARS-CoV OR SARS OR Severe acute respiratory syndrome OR nCoV OR Novel coronavirus*

**#2. Follow-up**

Long-COVID OR Post-acute COVID OR Post-COVID OR Follow-up OR Follow* OR Discharge* OR Recover* OR Surviv* OR Long-term OR Chronic OR Persistent OR Residual OR Sequel* OR Complication* OR Consequence*

**#3. Chest CT / Pulmonary function test**

MeSH descriptor: [Tomography, Emission-Computed] explode all trees

OR

MeSH descriptor: [Respiratory Function Tests] explode all trees

OR

Computed tomography OR CT OR Chest CT OR PFT OR Pulmonary function OR Respiratory function OR Lung function OR Spirometry OR Spirometer OR Diffusing capacity OR Diffusion capacity OR Diffusing OR Diffusion OR Transfer factor OR Transfer

**# Final search strategy: #1 AND #2 AND #3**

**Appendix 2 – Tools for assessing risk of bias in this study**

**1) External validity**

#1. Was the study’s target population a close representation of the national population in relation to relevant variables?

#2. Was the sampling frame a true or close representation of the target population?

#3. Was some form of random selection used to select the sample, or was a census undertaken?

#4. Was the likelihood of nonresponse bias minimal?

**2) Internal validity**

#5. Were data collected directly from the subjects (as opposed to a proxy)?

#6. Was an acceptable case definition used in the study?

#7. Was the study instrument that measured the parameter of interest shown to have validity and reliability?

#8. Was the same mode of data collection used for all subjects?

#9. Was the length of the shortest prevalence period for the parameter of interest appropriate?

#10. Were the numerator(s) and denominator(s) for the parameter of interest appropriate?

Reference: Hoy D, Brooks P, Woolf A, Blyth F, March L, Bain C, et al. Assessing risk of bias in prevalence studies: modification of an existing tool and evidence of interrater agreement. J Clin Epidemiol. 2012;65(9):934-9

**Figure S1.** Forest plot presenting the pooled estimate of percent predicted values for lung function parameters

A) Diffusion capacity for carbon dioxide (DLCO)

B) Forced vital capacity (FVC)

C) Total lung capacity (TLC)

**

**

**Figure S2.** Funnel plots presenting publication biases of studies on PFT abnormalities

A) Diffusion capacity for carbon dioxide (DLCO)

B) Forced vital capacity (FVC)

C) Total lung capacity (TLC)

**

**

**Figure S3.** Meta-regression plots presenting the effects of patient characteristics on the prevalence of reduced FVC

A) Age

B) Smoking history

C) Proportion of patients with severe COVID-19

D) Proportion of patients with critical COVID-19

**

**

**Figure S4.** Meta-regression plots presenting the effects of patient characteristics on the prevalence of reduced TLC

A) Age

B) Smoking history

C) Proportion of patients with severe COVID-19

D) Proportion of patients with critical COVID-19

**

**

**Figure S5.** Funnel plots presenting publication biases of studies on chest CT findings

A) Pulmonary fibrosis

B) Ground-glass opacity

C) Normal (complete resolution)

**

**

**Table S1. Summary of included studies**

| Author | Title | Journal | Study region | DOI |
| --- | --- | --- | --- | --- |
| Aparisi Á | Exercise Ventilatory Inefficiency in Post-COVID-19 Syndrome: Insights from a Prospective Evaluation | J Clin Med | Spain | 10.3390/jcm10122591 |
| Bardakci MI | Evaluation of long-term radiological findings, pulmonary functions, and health-related quality of life in survivors of severe COVID-19 | J Med Virol | Turkey | 10.1002/jmv.27101 |
| Bellan M | Long-term sequelae are highly prevalent one year after hospitalization for severe COVID-19 | Sci Rep | Italy | 10.1038/s41598-021-01215-4 |
| Caruso D | Post-Acute Sequelae of COVID-19 Pneumonia: Six-month Chest CT Follow-up | Radiology | Italy | 10.1148/radiol.2021210834 |
| Cassar MP | Symptom Persistence Despite Improvement in Cardiopulmonary Health - Insights from longitudinal CMR, CPET and lung function testing post-COVID-19 | EClinicalMedicine | United Kingdom | 10.1016/j.eclinm.2021.101159 |
| Chen Y | One-year follow-up of chest CT findings in patients after SARS-CoV-2 infection | BMC Med | China | 10.1186/s12916-021-02056-8 |
| Dai S | Follow-Up Study of the Cardiopulmonary and Psychological Outcomes of COVID-19 Survivors Six Months After Discharge in Sichuan, China | Int J Gen Med | China | 10.2147/IJGM.S337604 |
| Dorelli G | Importance of Cardiopulmonary Exercise Testing amongst Subjects Recovering from COVID-19 | Diagnostics | Italy | 10.3390/diagnostics11030507 |
| Faverio P | Six-Month Pulmonary Impairment after Severe COVID-19: A Prospective, Multicentre Follow-Up Study | Respiration | Italy | 10.1159/000518141 |
| Han X | Six-Month Follow-up Chest CT findings after Severe COVID-19 Pneumonia | Radiology | China | 10.1148/radiol.2021203153 |
| Hellemons ME | Persistent Health Problems beyond Pulmonary Recovery up to 6 Months after Hospitalization for COVID-19: A Longitudinal Study of Respiratory, Physical, and Psychological Outcomes | Ann Am Thorac Soc | Netherland | 10.1513/AnnalsATS.202103-340OC |
| Huang C | 6-month consequences of COVID-19 in patients discharged from hospital: a cohort study | Lancet | China | 10.1016/S0140-6736(20)32656-8 |
| Huang L | 1-year outcomes in hospital survivors with COVID-19: a longitudinal cohort study | Lancet | China | 10.1016/S0140-6736(21)01755-4 |
| Li Y | Follow-up study of pulmonary sequelae in discharged COVID-19 patients with diabetes or secondary hyperglycemia | Eur J Radiol | China | 10.1016/j.ejrad.2021.109997 |
| Liao T | Long-Term Effects of COVID-19 on Health Care Workers 1-Year Post-Discharge in Wuhan | Infect Dis Ther | China | 10.1007/s40121-021-00553-0 |
| Liu M | Follow-Up Study of the Chest CT Characteristics of COVID-19 Survivors Seven Months After Recovery | Front Med | China | 10.3389/fmed.2021.636298 |
| Liu T | Twelve-Month Systemic Consequences of Coronavirus Disease 2019 (COVID-19) in Patients Discharged From Hospital: A Prospective Cohort Study in Wuhan, China | Clin Infect Dis | China | 10.1093/cid/ciab703 |
| Milanese M | COVID-19 6 months after hospital discharge: pulmonary function impairment and its heterogeneity | ERJ Open Res | Italy | 10.1183/23120541.00196-2021 |
| Nabahati M | Post-COVID-19 pulmonary fibrosis and its predictive factors: a prospective study | Egypt J Radiol Nucl Med | Iran | 10.1186/s43055-021-00632-9 |
| Orzes N | A prospective evaluation of lung function at three and six months in patients with previous SARS-COV-2 pneumonia | Respir Med | Italy | 10.1016/j.rmed.2021.106541 |
| Pan F | Chest CT Patterns from Diagnosis to 1 Year of Follow-up in Patients with COVID-19 | Radiology | China | 10.1148/radiol.2021211199 |
| Safont B | Lung Function, Radiological Findings and Biomarkers of Fibrogenesis in a Cohort of COVID-19 Patients Six Months After Hospital Discharge | Arch Bronconeumol | Spain | 10.1016/j.arbres.2021.08.014 |
| Shah AS | Changes in pulmonary function and patient-reported outcomes during COVID-19 recovery: a longitudinal, prospective cohort study | ERJ Open Res | Canada | 10.1183/23120541.00243-2021 |
| Staudt A | Associations of Post-Acute COVID syndrome with physiological and clinical measures 10 months after hospitalization in patients of the first wave | Eur J Intern Med | Germany | 10.1016/j.ejim.2021.10.031 |
| Vijayakumar B | CT Lung Abnormalities after COVID-19 at 3 Months and 1 Year after Hospital Discharge | Radiology | United Kingdom | 10.1148/radiol.2021211746 |
| Wu Q | A Follow-Up Study of Lung Function and Chest Computed Tomography at 6 Months after Discharge in Patients with Coronavirus Disease 2019 | Can Respir J | China | 10.1155/2021/6692409 |
| Wu X | 3-month, 6-month, 9-month, and 12-month respiratory outcomes in patients following COVID-19-related hospitalisation: a prospective study | Lancet Respir Med | China | 10.1016/S2213-2600(21)00174-0 |
| Yan X | Follow-up study of pulmonary function among COVID-19 survivors 1 year after recovery | J Infect | China | 10.1016/j.jinf.2021.05.034 |
| Zhao Y | Follow-up study on COVID-19 survivors one year after discharge from hospital | Int J Infect Dis | China | 10.1016/j.ijid.2021.09.017 |
| Zhou F | Assessment of Sequelae of COVID-19 Nearly 1 Year After Diagnosis | Front Med | China | 10.3389/fmed.2021.717194 |

**Table S2. Summary of risk of bias evaluation**

| Study | Q1 | Q2 | Q3 | Q4 | Q5 | Q6 | Q7 | Q8 | Q9 | Q10 | Total score | Overall risk |
| --- | --- | --- | --- | --- | --- | --- | --- | --- | --- | --- | --- | --- |
| Aparisi Á | N | Y | Y | Y | Y | Y | Y | Y | Y | Y | 9 | low-to-moderate |
| Bardakci MI | N | N | Y | N | Y | Y | Y | Y | N | Y | 6 | moderate-to-high |
| Bellan M | N | Y | Y | N | Y | Y | Y | Y | Y | Y | 8 | low-to-moderate |
| Caruso D | N | N | Y | N | Y | Y | Y | Y | Y | Y | 7 | moderate-to-high |
| Cassar MP | N | Y | Y | N | Y | Y | Y | Y | Y | Y | 8 | low-to-moderate |
| Chen Y | N | Y | Y | N | Y | Y | Y | Y | Y | Y | 8 | low-to-moderate |
| Dai S | N | Y | Y | N | Y | N | Y | Y | N | Y | 6 | moderate-to-high |
| Dorelli G | N | Y | Y | N | Y | Y | Y | Y | N | Y | 7 | moderate-to-high |
| Faverio P | N | N | Y | N | Y | Y | Y | Y | N | Y | 6 | moderate-to-high |
| Han X | N | N | Y | N | Y | Y | Y | Y | N | Y | 6 | moderate-to-high |
| Hellemons ME | N | Y | Y | N | Y | Y | Y | Y | N | Y | 7 | moderate-to-high |
| Huang C | N | Y | Y | N | Y | Y | Y | Y | Y | Y | 8 | low-to-moderate |
| Huang L | N | Y | Y | N | Y | Y | Y | Y | Y | Y | 8 | low-to-moderate |
| Li Y | N | Y | Y | N | Y | Y | Y | Y | N | Y | 7 | moderate-to-high |
| Liao T | Y | Y | Y | N | Y | Y | Y | Y | Y | Y | 9 | low-to-moderate |
| Liu M | N | Y | Y | N | Y | Y | Y | Y | Y | Y | 8 | low-to-moderate |
| Liu T | N | Y | Y | N | Y | Y | Y | Y | Y | Y | 8 | low-to-moderate |
| Milanese M | N | N | Y | N | Y | Y | Y | Y | N | Y | 6 | moderate-to-high |
| Nabahati M | N | Y | Y | N | Y | Y | Y | Y | N | Y | 7 | moderate-to-high |
| Orzes N | N | Y | Y | N | Y | Y | Y | Y | N | Y | 7 | moderate-to-high |
| Pan F | N | Y | Y | N | Y | Y | Y | Y | Y | Y | 8 | low-to-moderate |
| Safont B | Y | N | Y | N | Y | Y | Y | Y | Y | Y | 8 | low-to-moderate |
| Shah AS | Y | Y | Y | N | Y | Y | Y | Y | N | Y | 8 | low-to-moderate |
| Staudt A | N | Y | Y | N | Y | Y | Y | Y | Y | Y | 8 | low-to-moderate |
| Vijayakumar B | N | Y | Y | N | Y | Y | Y | Y | Y | Y | 8 | low-to-moderate |
| Wu Q | N | Y | Y | N | Y | Y | Y | Y | N | Y | 7 | moderate-to-high |
| Wu X | N | N | Y | N | Y | Y | Y | Y | Y | Y | 7 | moderate-to-high |
| Yan X | Y | Y | Y | N | Y | Y | Y | Y | Y | Y | 9 | low-to-moderate |
| Zhao Y | Y | Y | Y | N | Y | Y | Y | Y | Y | Y | 9 | low-to-moderate |
| Zhou F | N | Y | Y | N | Y | Y | Y | Y | Y | Y | 8 | low-to-moderate |
